# Real-Life Experience on the Effect of SGLT2 Inhibitors vs. Finerenone vs. Combination on Albuminuria in Chronic Kidney Disease

**DOI:** 10.3390/diagnostics14131357

**Published:** 2024-06-26

**Authors:** Mohamad Hanouneh, Dustin Le, Bernard G. Jaar, Christina Tamargo, C. Elena Cervantes

**Affiliations:** 1 Division of Nephrology, Department of Medicine, Johns Hopkins University School of Medicine, Baltimore, MD 21287, USA; bjaar@jhmi.edu (B.G.J.); ctamarg1@jhmi.edu (C.T.); ccervan2@jhmi.edu (C.E.C.); 2 Nephrology Center of Maryland, Baltimore, MD 21239, USA; 3 Division of Nephrology, Thomas Jefferson University, Philadelphia, PA 19130, USA; dustin.le@jefferson.edu; 4 Welch Center for Prevention, Epidemiology, and Clinical Research, Baltimore, MD 21287, USA

**Keywords:** chronic kidney disease, albuminuria, SGLT2 inhibitors, finerenone

## Abstract

Background: There have been several recent advances in the care of patients with chronic kidney disease (CKD), including the use of sodium glucose cotransporter 2 (SGLT2) inhibitors and selective mineralocorticoid receptor antagonists (MRAs). There are very few data reporting the outcomes of these treatments in real-world experience. The aim of this retrospective study is to report the effects of SGLT2 inhibitors, finerenone, and their combination in CKD patients in our community-based setting. Methods: Ninety-eight patients with CKD with an estimated glomerular filtration rate (eGFR) between 25 and 90 mL/min per 1.73 m^2^ and a urine albumin-to-creatinine ratio (UACR) ≥ 30 mg/g were included. Patients were divided into three groups: two monotherapy groups of SGLT2 inhibitors or finerenone and a third combination group of therapy with SGLT2 inhibitors for the first 4 months and SGLT2 inhibitors and finerenone subsequently. The primary outcomes were the timing and percentage of patients achieving a >50% reduction in UACR from baseline. Results: Group 1 comprised 52 patients on SGLT2i, group 2 had 22 patients on finerenone, and group 3 had 24 patients on combination therapy. The baseline median UACR and mean eGFR were 513 mg/g and 47.9 mL/min per 1.73 m^2^ in group 1, 548.0 mg/g and 50.5 mL/min per 1.73 m^2^ in group 2, and 800 mg/g and 60 mL/min per 1.73 m^2^ in group 3. At baseline, 71 (72.4%) patients were on the angiotensin-converting enzyme inhibitor (ACEi) or the angiotensin receptor blocker (ARB), and 78 (79.5%) patients had type 2 diabetes. After 8 months of follow-up, a >50% decrease in albuminuria was achieved in 96% of patients in group 3, compared to 50% in group 1 and 59% in group 2 (*p*-values were <0.01 and <0.01, respectively). There was a statistically but not clinically significant change in mean potassium levels in group 2 (+0.4 mmol/L) compared to either group 1 (0.0 mmol/L with *p*-value: <0.01) or group 3 (−0.01 mmol/L with *p*-value: <0.01). However, there was no difference in potassium levels when comparing groups 1 and 3. At the end of the follow-up, the average difference in eGFR was −3.4 (8.8), −5.3(10.1), and −7.8 (11.2) mL/min per 1.73 m^2^ in groups 1, 2, and 3, respectively, without a statistically significant difference between groups. Conclusions: In this real-world experience in our community setting, the combination of SGLT2 inhibitors and finerenone in our adult patients with CKD was associated with a very significant and clinically relevant reduction in UACR, without an increased risk of hyperkalemia. Combination therapy of SGLT2 inhibitor and finerenone regarding background use of ACEi/ARB is feasible and should be encouraged for further albuminuria reductions in CKD patients.

## 1. Introduction

Angiotensin-converting enzyme inhibitors (ACEis) and angiotensin receptor blockers (ARBs) have long been the cornerstone of slowing the progression of chronic kidney disease (CKD). Despite their proven benefits, the risk of kidney failure remains substantial. Recent therapeutic advancements have introduced new mechanisms for slowing the progressive decline in kidney function by targeting specific pathways such as restoring the tubuloglomerular feedback mechanism or reducing inflammatory processes within the kidneys [1,2,3,4,5].

Sodium glucose co-transporter 2 (SGLT2) inhibitors restore the tubuloglomerular feedback and have demonstrated significant reductions in estimated Glomerular Filtration Rate (eGFR) decline [1,2]. The CREDENCE trial initially showed these benefits in patients with type 2 diabetes and CKD [1]. Subsequent trials, DAPA-CKD and EMPA-KIDNEY, extended these findings, showing significant reductions in the composite kidney endpoint with dapagliflozin and empagliflozin, respectively, independent of diabetes status [2,3]. EMPA-KIDNEY also showed enhanced risk reduction in patients with albuminuria in the absence of diabetes [3]. In DAPA-CKD, dapagliflozin yielded a notable decrease in the urine albumin-to-creatinine ratio (UACR) compared to a placebo [4].

Mineralocorticoid receptor antagonists (MRAs), such as finerenone, alleviate the inflammatory processes within the kidneys and constitute another effective class of medications that reduce albuminuria in patients with CKD. In FIDELIO-DKD, finerenone exhibited a significant reduction in UACR compared to a placebo [5]. FIDELIO-DKD and FIGARO-DKD trials demonstrated that finerenone significantly decreased the risk of a composite kidney outcome (kidney failure, sustained decline of ≥40% in the eGFR, or renal death) in patients with type 2 diabetes and CKD [5,6].

Despite the documented clinical advantages of these therapies in randomized controlled trials, some patients still progress toward kidney failure, especially those with persistent and higher albuminuria [7]. Post-hoc analyses from trials showed that combining SGLT2 inhibitors with conventional MRAs or finerenone provides an independent and complementary nephroprotective effect [8,9]. The results of the ROTATE-3 trial indicated that combining dapagliflozin and eplerenone led to a substantial and durable decrease in UACR levels within 4 weeks of treatment. Nevertheless, further research is necessary to ascertain the long-term benefits and risks associated with this combination therapy in a community setting [10]. Furthermore, it has not been reported whether the combination of the SGLT2 inhibitor and finerenone treatment, when compared to either treatment alone, leads to a further reduction in albuminuria in real-world settings over a longer period of follow-up.

KDIGO 2024 guidelines suggest using selective non-steroidal MRA, finerenone, for diabetic kidney disease in patients with eGFR > 25 mL/min per 1.73 m^2^ and UACR ≥ 30 mg/g [11]. These same guidelines recommend starting SGLT2i for patients with CKD with eGFR ≥ 20 mL/min per 1.73 m^2^ and UACR ≥ 200 mg/g [11]. 

Acknowledging the amplified advantages of combining SGLT2 inhibitors and selective MRAs in mitigating CKD progression, particularly in patients on renin–angiotensin–aldosterone system (RAAS) inhibitors, we conducted a retrospective analysis utilizing real-world data to evaluate their effectiveness in reducing albuminuria, a key marker of CKD progression, and its impact on the risk of hyperkalemia. Our focus was on examining the effects of SGLT2 inhibitors alone, finerenone alone, and the combined use of SGLT2 inhibitors with finerenone in patients from our established CKD clinic.

## 2. Study Design and Patients

We conducted a retrospective analysis involving patients from our specialty CKD clinic with an eGFR ranging from 25 to 90 mL/min per 1.73 m^2^ and UACR ≥ 30 mg/g. The patients were categorized into three groups with an 8-month follow-up: group 1 was treated with SGLT2 inhibitors alone, group 2 was treated with finerenone alone, and group 3 was treated with combination therapy—but with SGLT2i inhibitors for the first 4 months and then finerenone was subsequently added. The primary outcome measure was the percentage of patients achieving a >50% reduction in UACR from their baseline levels. We reported the percentage of patients achieving >30% and >40% reductions in UACR from their baseline levels as part of a sensitivity analysis. Additional outcomes included the percentage of reduction in albuminuria at the end of the 8-month follow-up period, changes in serum potassium levels, and changes in eGFR.

## 3. Statistical Analysis

We used one-way analysis of variance, Kruskal–Wallis, and Chi-square tests, as is appropriate with post-hoc testing, to compare baseline demographics between participants (SGLT2 inhibitors vs. finerenone vs. combination SGLT2 inhibitors + finerenone). We report the proportion of patients achieving a 50% reduction in UACR from baseline with each treatment. Due to limited power, we report Fisher’s exact test *p*-value comparing these outcomes (comparing all 3 medications and 1:1 post-hoc comparisons). We then estimated the percent of reduction in UACR by treatment group using linear regression with adjustments for baseline age, eGFR, ACEi/ARB use, diabetes status, and albuminuria. The percent of reduction in UACR and albuminuria were transformed into natural logarithms due to skewed distributions. We then repeated this analysis by treatment group looking at two other outcomes: changes in potassium and changes in eGFR. We used the same covariates as above, as well as the corresponding baseline value (i.e., baseline potassium or baseline eGFR). We also report the number of patients with a potassium >5 mmol/L at any time during the follow-up period and the Fisher-exact *p*-value across treatments. Statistical analyses were conducted using R version 4.2. Results with a two-tailed *p*-value of <0.05 were considered statistically significant.

## 4. Results

### 4.1. Baseline Characteristics

Out of the 402 CKD patients screened between January 2022 and September 2023, a total of 98 patients met the inclusion criteria and were included in this retrospective study. Among those, 52 (53%) patients in group 1 were on SGLT2 inhibitors (dapagliflozin 10 mg daily, empagliflozin 10 mg daily, or empagliflozin 25 mg daily), 22 (22.4%) patients in group 2 were on finerenone 10 mg or 20 mg daily, while 24 (24.4%) patients in group 3 were on SGLT2 inhibitors (dapagliflozin 10 mg daily, empagliflozin 10 mg daily, or empagliflozin 25 mg daily) for the initial 4 months, followed by a combination of SGLT2 inhibitors and finerenone 10 mg or 20 mg daily. The baseline demographics, as well as the clinical and biochemical characteristics of the three groups, are presented in Table 1. For all patients, the average eGFR was 51.0 mL/min per 1.73 m^2^ and the median UACR was 580 mg/g. At baseline, 71 (72.4%) patients were on ACEi/ARB for at least 3 months before starting SGLT2 inhibitors or finerenone, and 78 (79.5%) patients had type 2 diabetes. For patients in the SGLT2 inhibitors group, the baseline median UACR was 513 mg/g (interquartile range (IQR) of 107.0 to 1072.5) compared to 548.0 mg/g (IQR of 265.0 to 1559.0) in the finerenone group and 800 mg/g (IQR of 443.0 to 2684.5) in the combination group.

### 4.2. Effects on Albuminuria

**Figure 1:**
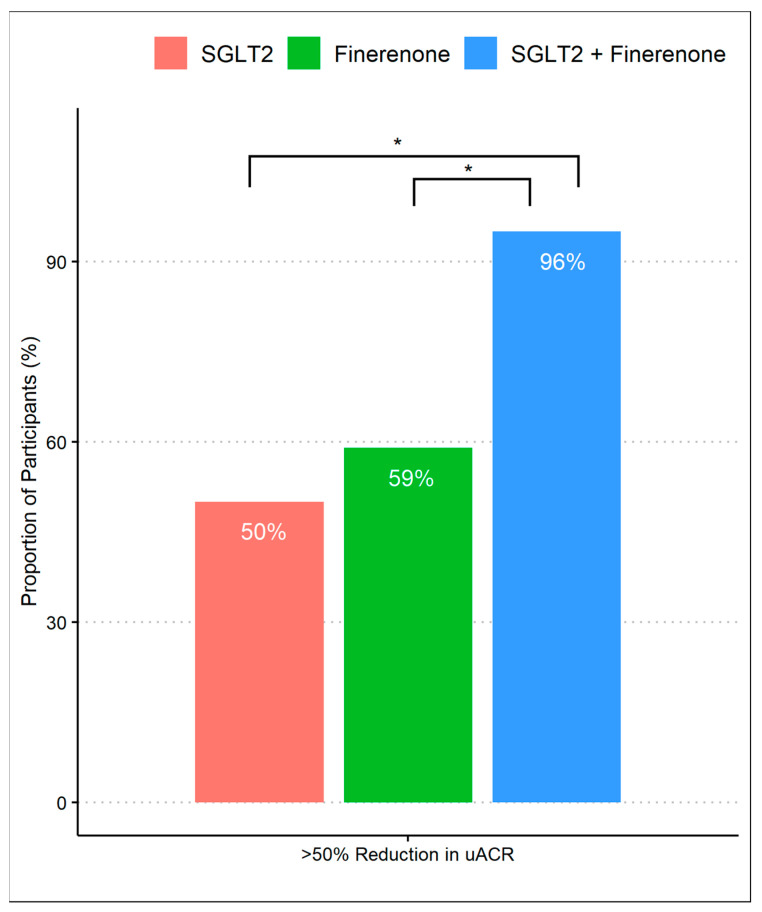
Proportion of patients achieving >50% reduction in UACR during treatment with SGLT2 inhibitor monotherapy, finerenone monotherapy, and combination of SGLT2 inhibitors and finerenone. * *p*-value of <0.01 with Fisher’s exact test with post-hoc pairwise comparisons.

**Figure 2:**
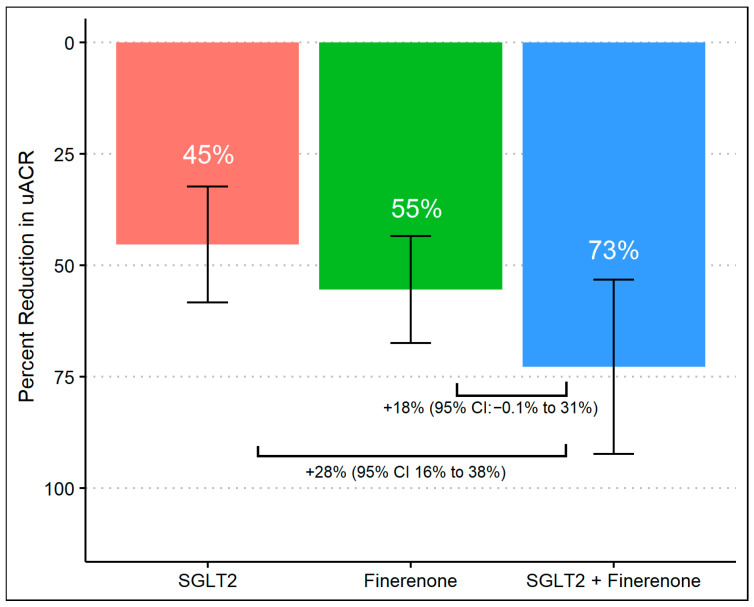
Percent change in UACR from baseline during treatment with SGLT2 inhibitor monotherapy, finerenone monotherapy, and combination of SGLT2 inhibitors and finerenone with multivariable linear regression.

After 8 months of follow-up, 63% achieved a >50% decrease in UACR, which differed according to the treatment group, with group 3 achieving a higher proportion (96%) compared to group 1 (50%) and group 2 (59%) (*p*-values were <0.01 and <0.01, respectively). There was no difference in primary outcomes between group 1 (SGLT2 inhibitor alone) and group 2 (finerenone alone) (Figure 1). When we assessed the reduction in geometric mean UACR, there was a significant difference comparing group 1 (45%, 95% CI 32% to 58%) to group 3 (73%, 95% CI 53% to 92%) with a difference reduction of 28% (95% Cl: 16% to 38%) and a *p*-value of < 0.01. Despite observing a substantial reduction in albuminuria in group 3 (73%, 95% CI 53% to 92%) compared to group 2 (55%, 95% CI 43% to 67%), the difference was 18% (95% Cl: −0.1% to 31%) and did not reach statistical significance (*p*-value 0.07). Additionally, there was no significant difference comparing the percent of albuminuria reduction between group 1 and group 2. (Figure 2). 

### 4.3. Effects on eGFR

The baseline mean (standard deviation) eGFR across groups was 47.9 (19.0) mL/min per 1.73 m^2^ in group 1, 50.5 (19.5) in group 2, and 60 (21.5) in group 3. At the end of the follow-up period (8 months), eGFR was 44.5 (18.7) mL/min per 1.73 m^2^, 45.2 (16.3), and 52.1 (24.2), respectively. The average difference was −3.4 (8.8), −5.3(10.1), and −7.8 (11.2) for groups 1, 2, and 3, respectively, without a statistically significant difference between groups. Analyses were adjusted for baseline eGFR, and covariates were unchanged across the three groups. 

### 4.4. Effects on Serum Potassium

**Figure 3:**
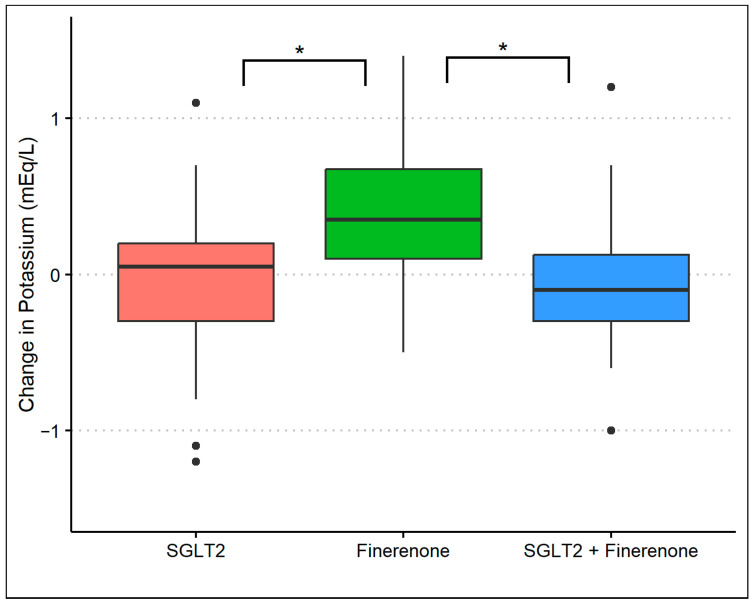
Changes in serum potassium during treatment with SGLT2 inhibitor monotherapy, finerenone monotherapy, and the combination of SGLT2 inhibitors and finerenone. * *p*-value of <0.01 with multivariable linear regression.

The baseline mean (standard deviation) serum potassium level was 4.5 (0.4) mmol/L in group 1, 4.3 (0.5) mmol/L in group 2, and 4.4 (0.5) mmol/L in group 3. At the 8-month follow-up, the serum potassium was 4.4 (0.4) mmol/L in group 1, 4.7 (0.4) mmol/L in group 2, and 4.3 (0.5) mmol/L in group 3 (*p*-value: 0.01) with an average difference of 0.0 (0.04), 0.4 (0.5), and −0.10 (0.5), respectively (*p*-value: <0.01). After adjusting for baseline potassium and baseline covariates, there was a significant difference in potassium levels in group 2 compared to either group 1 (*p*-value: <0.01) or group 3 (*p*-value: <0.01). However, there was no difference in potassium levels comparing groups 1 and 3 (Figure 3). During the observation period, nine patients had a potassium level of >5.0 mmol/L at least once (three patients in group 1, five patients in group 2, and one patient in group 3) with no clinical consequence.

### 4.5. Sensitivity Analysis

When we looked at the outcome of a 40% reduction in UACR, all patients in group 3 achieved a 40% reduction in albuminuria vs. 69% in group 1 and 82% in group 2 (*p*-values of < 0.01 and 0.04, respectively) (Figure S1). The results comparing a 30% reduction in UACR were grossly similar except there was no longer a statistically significant difference between groups 2 and 3 (91% and 100%, respectively) (Figure S1).

## 5. Discussion

This report represents one of the few studies shedding light on kidney-related outcomes in patients receiving treatment with the newer agents aimed at slowing CKD progression.

Prescribing SGLT2 inhibitors and finerenone can pose several challenges for healthcare providers. Firstly, there may be limitations in insurance coverage and formulary restrictions, which can impact the accessibility of these medications for patients. Additionally, prescribing these drugs requires a thorough assessment of the patient’s kidney function, comorbidities, and potential drug interactions. Although the ROTATE-3 trial indicated that combining dapagliflozin and eplerenone led to a substantial and durable decrease in UACR levels within 4 weeks of treatment, in clinical practice, the simultaneous prescription of SGLT2 inhibitors and finerenone is uncommon at this time [10]. The sequential addition of medications such as SGLT2 inhibitors followed by finerenone, or vice versa, is a more common strategy employed in the management of CKD. This sequential approach allows healthcare providers to gauge the individual response and tolerability to each medication before introducing another. Typically, SGLT2 inhibitors are initiated first due to their established benefits and side-effect profile. Following an assessment of the patient’s response, tolerability, and achievement of treatment goals, finerenone may be added to further decrease albuminuria and provide additional kidney and cardiovascular protection. Alternatively, in some cases, finerenone may be initiated first, particularly in patients with contraindications or intolerance to SGLT2 inhibitors. This stepwise approach enables personalized treatment adjustments based on the patient’s clinical status and ensures the judicious use of medications to achieve optimal outcomes in the management of CKD and minimize side-effects.

Our goal in this study is to show the real-world experience and benefits of using SGLT2 inhibitors and finerenone in CKD patients with proteinuria on ACEi or ARB. In this retrospective analysis involving individuals with CKD and albuminuria, combination therapy with SGLT2 inhibitors and finerenone (vs. SGLT2 inhibitors monotherapy or finerenone monotherapy) was well-tolerated and resulted in a significant reduction in albuminuria, demonstrating their synergistic effect. Combination therapy seemed to have reduced the risk of hyperkalemia compared to finerenone use alone, with no significant change observed in eGFR compared to either SGLT2 inhibitors or finerenone monotherapy groups. These findings advocate for prospective trials in CKD patients to validate the long-term safety and effectiveness of combination therapy with SGLT2 inhibitors and selective MRAs in improving clinical outcomes.

SGLT2 inhibitors and MRAs may complement each other, improving kidney outcomes through independent mechanisms. Studies in rats showed that combining empagliflozin and finerenone had a synergistic anti-albuminuric effect, enhancing survival [12]. Trials like DAPA-CKD and FIDELIO-DKD demonstrated the positive effects of dapagliflozin and finerenone regardless of MRA or SGLT2 inhibitor use at baseline, respectively. Refs. [8,9] Combining dapagliflozin and eplerenone in the ROTATE-3 trial showed a significant UACR reduction, suggesting a strong synergistic effect in reducing albuminuria [10]. Our findings are in line with these preliminary reports supporting this combination therapy.

Combining SGLT2 inhibitors with MRAs may raise safety concerns, particularly regarding hyperkalemia risk. MRAs can exacerbate hyperkalemia, especially in CKD patients on ACEis or ARBs. SGLT2 inhibitors enhance potassium secretion, potentially minimizing this risk [13,14]. Meta-analyses and trial data suggest a modest decrease in hyperkalemia risk with SGLT2 inhibitors when combined with MRAs [15,16]. In our study, the combination of SGLT2 inhibitors with finerenone decreased the risk of hyperkalemia compared to finerenone monotherapy.

Both SGLT2 inhibitors and finerenone have hemodynamic effects in the glomeruli that initially reduce the eGFR. Our observations found that combining SGLT2 inhibitors with finerenone resulted in a greater reduction in eGFR, though this difference was not statistically significant (in our smaller sample size study) compared to SGLT2 inhibitors alone or finerenone alone. Since the effects of combination therapy on kidney function are synergistic, a more substantial reduction in eGFR is expected, which raises safety concerns. However, it is crucial to emphasize that these agents reduce glomerular hyperfiltration, a mechanism whose management is associated with the long-term stabilization of kidney function [17,18].

This study has several limitations. A significant limitation is the small sample size and the single-center nature of our cohort. Nonetheless, the practice pattern to slow the progression of CKD is currently evidence-based and guidelines-driven, where interventions are similar among practicing nephrologists, which is not different from the collective experience of the five nephrologists in our specialty practice. To assess the long-term benefit of our intervention, participants were only enrolled if they were followed for 8 months of therapy with available UACR at baseline and at the end of the follow-up period. It is also essential to recognize that other factors not considered may have influenced the changes in albuminuria during the follow-up period. For instance, we did not explore improvements in blood pressure or glycemic control in diabetic patients, nor changes in weight. In addition, given the retrospective nature of this study, residual confounding is likely. There could also be confounding by the indication where healthier people received combination therapy instead of monotherapy. Further reports on larger and extended prospective observations in real-world settings are essential to validate our findings and support the combination therapy of SGLT2 inhibitors and MRA in CKD patients receiving RAAS blockade agents.

In summary, the combined administration of SGLT2 inhibitors and finerenone (vs. SGLT2 inhibitors alone or finerenone alone) in patients receiving or not receiving RAAS blockade agents demonstrated a greater reduction in UACR levels among CKD patients and minimized the risk of hyperkalemia compared with the finerenone group alone. These findings confirm that the combined use of SGLT2 inhibitors and finerenone can further impede the progression of kidney disease in patients with CKD, emphasizing the need for validation through a large-scale prospective clinical trial.

## Figures and Tables

**Table 1:** The baseline demographics and clinical and biochemical characteristics.

	Group 1: SGLT2i *N* = 52	Group 2: Finerenone *N* = 22	Group 3: SGLT2i/Finerenone *N* = 24	*p*-Value
Age, years	71 [62, 76]	72 [67, 75]	69 [65, 75]	0.60 *
Female	23 (44%)	10 (45%)	4 (17%)	0.15 **
Male	29 (56%)	12(55%)	20 (83%)	
Type 2 Diabetes	38 (73%)	18 (82%)	22 (92%)	0.17 **
ACEi or ARB use	34 (65%)	18 (82%)	19 (79%)	0.25 **
Serum potassium, mmol/L	4.5 (0.4)	4.3 (0.5)	4.4 (0.5)	0.41 ***
UACR, mg/g	513 [107, 1073] ^a^	548 [265, 1559]	800 [443, 2685] ^b^	0.05 *
EGFR, mL/min per 1.73 m^2^	48 (19) ^a^	51 (20)	60 (22)^b^	0.05 ***
Serum sodium, mEq/L	140 (3)	140 (4)	139 (4)	0.64 ***
Serum Hemoglobin, g/dL	12.5 (1.8)	12.8 (2.1)	12.2 (2.0)	0.51 ***

Data are reported as mean (SD), number (percent), or median (interquartile interval). Letters in each row indicate statistical differences at *p*-value of <0.05 in post-hoc testing. * Krukal–Wallis test with post-hoc Dunn test; ** Chi-square; *** ANOVA with Tukey’s post-hoc test. Abbreviations: ACEi: angiotensin-converting enzyme inhibitor, ARB: angiotensin receptor blocker, UACR: urine urinary albumin-to-creatinine ratio, EGFR: estimated Glomerular Filtration Rate.

## Data Availability

The raw data supporting the conclusions of this article will be made available by the authors on request.

## Supplementary Materials

The following supporting information can be downloaded at: https://www.mdpi.com/article/10.3390/diagnostics14131357/s1, Figure S1: Proportion of patients achieving a >30%, >40%, and >50% reduction in UACR during treatment with SGLT2 inhibitors monotherapy, finerenone monotherapy, and combination of SGLT2 inhibitors and finerenone. * *p*-value of <0.05 with Fisher’s exact test with post-hoc pairwise comparison. ** *p*-value of <0.01 with Fisher’s exact test with post-hoc pairwise comparison.

## References

[1] Perkovic V., Jardine M.J., Neal B., Bompoint S., Heerspink H.J.L., Charytan D.M., Edwards R., Agarwal R., Bakris G., Bull S. (2019). CREDENCE Trial Investigators: Canagliflozin and renal outcomes in type 2 diabetes and nephropathy. N. Engl. J. Med..

[2] Heerspink H.J.L., Stefánsson B.V., Correa-Rotter R., Chertow G.M., Greene T., Hou F.F., Mann J.F.E., McMurray J.J.V., Lindberg M., Rossing P. (2020). DAPA-CKD Trial Committees and Investigators: Dapagliflozin in patients with chronic kidney disease. N. Engl. J. Med..

[3] Herrington W.G., Staplin N., Wanner C., Green J.B., Hauske S.J., Emberson J.R., Preiss D., Judge P., Mayne K.J., The EMPA-KIDNEY Collaborative Group (2023). Empagliflozin in Patients with Chronic Kidney Disease. N. Engl. J. Med..

[4] Jongs N., Greene T., Chertow G.M., McMurray J.J.V., Langkilde A.M., Correa-Rotter R., Rossing P., Sjöström C.D., Stefansson B.V., Toto R.D. (2021). Effect of dapagliflozin on urinary albumin excretion in patients with chronic kidney disease with and without type 2 diabetes: A prespecified analysis from the DAPA-CKD trial. Lancet Diabetes Endocrinol..

[5] Bakris G.L., Agarwal R., Anker S.D., Pitt B., Ruilope L.M., Rossing P., Kolkhof P., Nowack C., Schloemer P., Joseph A. (2020). FIDELIO-DKD Investigators: Effect of finerenone on chronic kidney disease outcomes in type 2 diabetes. N. Engl. J. Med..

[6] Pitt B., Filippatos G., Agarwal R., Anker S.D., Bakris G.L., Rossing P., Kolkhof P., Nowack C., Gebel M., Ruilope L.M. (2021). FIGARO-DKD Investigators: Cardiovascular events with finerenone in kidney disease and type 2 diabetes. N. Engl. J. Med..

[7] Oshima M., Neuen B.L., Li J., Perkovic V., Charytan D.M., de Zeeuw D., Edwards R., Greene T., Levin A., Mahaffey K.W. (2020). Early change in albuminuria with canagliflozin predicts kidney and cardiovascular outcomes: A *post hoc* analysis from the CREDENCE Trial. J. Am. Soc. Nephrol..

[8] Rossing P., Filippatos G., Agarwal R., Anker S.D., Pitt B., Ruilope L.M., Chan J.C.N., Kooy A., McCafferty K., Schernthaner G. (2021). FIDELIO-DKD Investigators: Finerenone in predominantly advanced CKD and type 2 diabetes with or without sodium-glucose cotransporter-2 inhibitor therapy. Kidney Int. Rep..

[9] Provenzano M., Jongs N., Vart P., Stefánsson B.V., Chertow G.M., Langkilde A.M., McMurray J.J.V., Correa-Rotter R., Rossing P., Sjöström C.D. (2021). The kidney protective effects of the sodium–glucose cotransporter-2 inhibitor, dapagliflozin, are present in patients with chronic kidney disease treated with mineralocorticoid receptor antagonists. Kidney Int. Rep..

[10] Provenzano M., Puchades M.J., Garofalo C., Jongs N., D’Marco L., Andreucci M., De Nicola L., Gorriz J.L., Heerspink H.J.L., ROTATE-3 Study Group (2022). Albuminuria-Lowering Effect of Dapagliflozin, Eplerenone, and Their Combination in Patients with Chronic Kidney Disease: A Randomized Crossover Clinical Trial. J. Am. Soc. Nephrol..

[11] Group KDIGOKCW (2024). KDIGO 2024 Clinical Practice Guideline for the Evaluation and Management of Chronic Kidney Disease. Kidney Int..

[12] Kolkhof P., Hartmann E., Freyberger A., Pavkovic M., Mathar I., Sandner P., Droebner K., Joseph A., Hüser J., Eitner F. (2021). Effects of finerenone combined with empagliflozin in a model of hypertension-induced end-organ damage. Am. J. Nephrol..

[13] Palmer B.F. (2015). Regulation of potassium homeostasis. Clin. J. Am. Soc. Nephrol..

[14] Karet F.E. (2009). Mechanisms in hyperkalemic renal tubular acidosis. J. Am. Soc. Nephrol..

[15] Neuen B.L., Oshima M., Agarwal R., Arnott C., Cherney D.Z., Edwards R., Langkilde A.M., Mahaffey K.W., McGuire D.K., Neal B. (2022). Sodium-glucose cotransporter 2 inhibitors and risk of hyperkalemia in people with type 2 diabetes: A meta-analysis of individual participant data from randomized controlled trials [published online ahead of print 8 April 2022]. Circulation.

[16] Agarwal R., Joseph A., Anker S.D., Filippatos G., Rossing P., Ruilope L.M., Pitt B., Kolkhof P., Scott C., Lawatscheck R. (2022). FIDELIO-DKD Investigators: Hyperkalemia risk with finerenone: Results from the FIDELIO-DKD Trial. J. Am. Soc. Nephrol..

[17] Holtkamp F.A., de Zeeuw D., Thomas M.C., Cooper M.E., de Graeff P.A., Hillege H.J., Parving H.-H., Brenner B.M., Shahinfar S., Heerspink H.J.L. (2011). An acute fall in estimated glomerular filtration rate during treatment with losartan predicts a slower decrease in long-term renal function. Kidney Int..

[18] Oshima M., Jardine M.J., Agarwal R., Bakris G., Cannon C.P., Charytan D.M., de Zeeuw D., Edwards R., Greene T., Levin A. (2021). Insights from CREDENCE trial indicate an acute drop in estimated glomerular filtration rate during treatment with canagliflozin with implications for clinical practice. Kidney Int..

