# Supplementary material for: Real-Life Experience on the Effect of SGLT2 Inhibitors vs. Finerenone vs. Combination on Albuminuria in Chronic Kidney Disease
Source: Diagnostics (Basel). 2024 Jun 26;14(13):1357. doi: 10.3390/diagnostics14131357 (PMC11241372; doi:10.3390/diagnostics14131357)
Supplement: Supplementary file 1 [file diagnostics-14-01357-s001.zip › diagnostics-3071066-supplementary.pdf]

diagnostics

Supplementary Materials

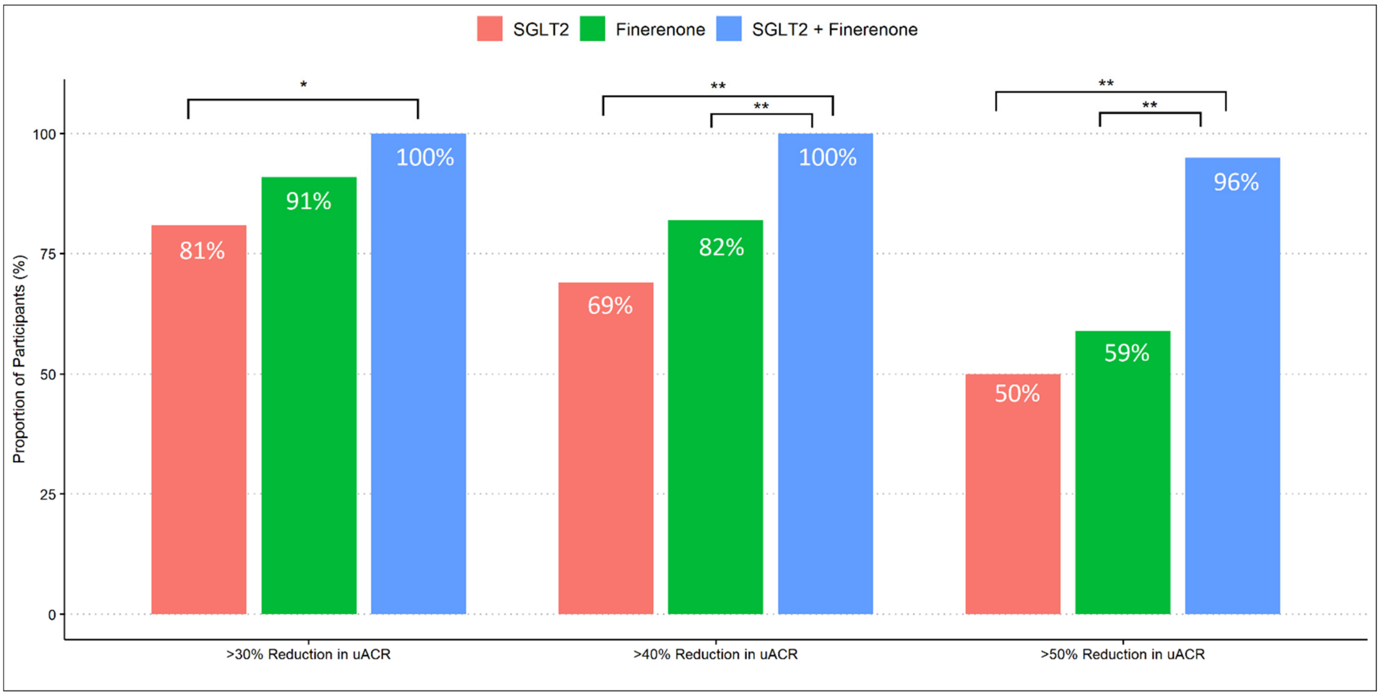

Figure S1. Proportion of patients for achieving a >30%, >40% and >50% reduction in UACR during treatment with SGLT2 inhibitors monotherapy, finerenone monotherapy and combination SGLT2 inhibitors-finerenone. * p-value <0.05 with Fisher's exact test with post-hoc pairwise comparison. ** p-value <0.01 with Fisher's exact test with post-hoc pairwise comparison.
